# Pilot Translational Precision Biobehavioral Assays for Early Detection of Motor Impairments in a Rat Model of Cerebral Palsy

**DOI:** 10.3390/life13081746

**Published:** 2023-08-14

**Authors:** Gwendolyn Gerner, Vera Joanna Burton, Yuma Kitase, Shenandoah Robinson, Lauren L. Jantzie

**Affiliations:** 1 Department of Neuropsychology, Kennedy Krieger Institute, Baltimore, MD 21205, USA; 2 Department of Psychiatry and Behavioral Sciences, Johns Hopkins University School of Medicine, Baltimore, MD 21205, USA; 3 Department of Neurology and Developmental Medicine, Kennedy Krieger Institute, Baltimore, MD 21205, USAsrobin81@jhmi.edu (S.R.); ljantzie@jhmi.edu (L.L.J.); 4 Department of Neurology, Johns Hopkins University School of Medicine, Baltimore, MD 21205, USA; 5 Department of Pediatrics, Division of Perinatal-Neonatal Medicine, Johns Hopkins University School of Medicine, Baltimore, MD 21205, USA; 6 Department of Neurosurgery, Johns Hopkins University School of Medicine, Baltimore, MD 21205, USA

**Keywords:** translational methods, cerebral palsy, infant motor movements, general movements assessment, early detection, rats, preclinical

## Abstract

Background: Cutting-edge neonatal programs diagnose cerebral palsy (CP) or “high risk of CP” using validated neurobehavioral exams in combination with risk history and neuroimaging. In rat models, digital gait analyses are the gold standard adult assessment, but tools in infant rats are limited. Refinement of infant rat neurobehavioral correlates of CP will establish translational behavioral biomarkers to delineate early mechanisms of CP in both humans and rodent models of CP. Objective: To facilitate precision medicine approaches of neurodevelopmental health and integrate basic and clinical research approaches for CP, we developed and piloted a new assay of neonatal rat neurobehavior to mimic human neonate exams. Methods: Our established rat model of CP secondary to chorioamnionitis (CHORIO) that induces bilateral motor impairment reminiscent of spastic CP was used. On postnatal day 10 (P10), 5 min videos were recorded of 26 (6 sham and 20 CHORIO) animals moving freely in a cage were reviewed by an evaluator trained in the human General Movements Assessment (GMA). Non-blinded observation revealed two behaviors that differed between rat pups in each group (time spent rearing; multi-dimensional nose sweeping; and sniffing). Each video was re-coded for these criteria by an evaluator blind to group status. Differences between sham and CP groups were analyzed using a Mann–Whitney U-test or Student’s *t*-test (*p* < 0.05 level of significance). Results: Neonatal rats with CP exhibited sensorimotor impairment and decreased spatial exploration. CP rats spent significantly less time rearing (17.85 ± 1.60 s vs. 34.8 ± 2.89 s, *p* = 0.007) and engaged in multi-dimensional nose sweeping and sniffing (2.2 ± 0.58 episodes vs. 5.5 ± 0.96 episodes, *p* = 0.03) than sham controls. Conclusions: These pilot findings of harmonized translational and precision biobehavioral assays provide an opportunity for increased expediency of clinical trials at the earliest stages of brain development.

## 1. Introduction

Cutting-edge neonatal neurodevelopmental follow-up programs utilize empirically validated tools to support early detection of cerebral palsy (CP) [1,2,3,4,5]. Functional motor examinations and standardized neurologic exam in combination with risk history and neuroimaging can be used to accurately predict Cerebral Palsy in infancy. One tool recommended as part of the early diagnosis algorithm is Prechtl’s General Movement Assessment (GMA) [1,2]. The GMA, which has a high predictive power for early detection of CP, is a diagnostic tool based on visual inspection of specific underlying motor movements [3,4,5,6,7]. Specifically, the examiner evaluates the quality and type of writhing motor movements and the presence/absence and the quality of fidgety motor movements in the human infant [6,7]. These motor movements are spontaneously generated motor movements involving the whole body that present in a variable sequence and evolve over the first 5 months of the human infant’s life [6].

Human infant writhing movements occur from 10 weeks of fetal life to 10 weeks post-term age equivalent [6,8,9]. Normal writhing movements are fluid and multiplane movements that run through the entire body and occur with variable intensity, speed, and direction. Infants who have a risk of neurological injury due to preterm birth, hypoxic–ischemic encephalopathy, perinatal stroke and other potential prenatal/perinatal insults are at a higher risk for abnormal writhing movements [6,8,9]. Abnormal writing movements are categorized as poor repertoire, cramp synchronized, or chaotic [6]. Poor repertoire is the presence of writing movements that are of poorer quality with a low frequency and less variability, while cramped synchronized movements are rigid movements with simultaneous contraction of the extremities. These movements are predictable and occur with little variation [6]. Cramped synchronized movements are associated with a higher risk of CP [6,8,9]. Chaotic movements are rare, but are characterized by large amplitude movements without fluidity or smoothness [6]. These movements are also more concerning for a higher risk of CP [6,8,9].

Human infant fidgety movements occur between ages 3 and 5 months (corrected degree for prematurity if applicable) [7]. These movements are thought to occur as the body is calibrating proprioceptive feedback in order to begin to refine volitional movements. Normal fidgety movements are high frequency and low amplitude movements occurring at distal and proximal joints and are reassuring for normal motor development when seen in at least three limbs. Abnormal fidgety movements are fidgety movements with exaggerated amplitude, speed, and jerkiness [7]. While abnormal fidgety movements may be associated with neurologic deficit, they have low prediction value. Absence of fidgety movements, on the other hand, is highly predictive of neurological impairment, and specifically of CP [3,6,7].

The combination of cramped synchronized motor movements in the writhing stage and then later absent fidgety movements is highly predictive of a diagnosis of cerebral palsy in human infants [1]. This is one clinical tool that aides in early risk stratification and identification for a diagnosis of CP. Currently, early detection of high risk for CP or a diagnosis of CP currently expedites access to CP-specific interventions that can decrease the later severity of motor impairments associated with CP; however, there is an urgent need for the development of pharmacological interventions for neonates at high risk of neurological injury and CP, and ongoing translational neuroscience is focusing on treatments to ameliorate risk or target interventions even earlier. Furthermore, clinical trials for promising pharmacological interventions are translational.

Presently, the gold standard approach to identifying motor deficits akin to CP in adult rats (i.e., P25–P26) utilizes computer digital gait analysis [10,11,12,13,14], and this is a rigorous approach for evaluating responses to pharmacological interventions in the early stages of clinical trials [15,16]. Digigait software analyzes multiple gait metrics and kinematic measurements based on the position of the paws, amount of paw contact, and time of each paw step. Additionally, posture, cadence, stance duration, braking, swing, and propulsion phases of each step are measured. This technique provides valuable information about changes in gait patterns in adult rats who had a prenatal injury induced with different lesion types [10,11,12,13,14]. Differences in gait that have been previously observed following prenatal hypoxic–ischemic and infectious inflammatory lesions and include difficulty with coordinated stepping or reduced stride length, more variation in the length of stride, increased stride frequency, and ataxia [14]. Digigait is able to detect specific types of gait patterns that approximate those observed in humans who have CP, such as toe walking and scissor gait [10,15,16]. Despite the precise information that can be provided by digital gait analysis in adult rats, there are no precise metrics of motor impairment in rats at ages equivalent to human infants, limiting cross comparisons between animal and human studies.

Lack of harmonization between human clinical methods and animal methods limits the translation necessary for clinical trials of promising pharmacological interventions for neurologic injury and CP, and precise translational biobehavioral assays capturing healthy and abnormal state-trait domains are urgently needed. Therefore, the goal of the current study was to facilitate development of a translational methodology that could be readily utilized to detect neurologic injury leading to CP versus neurological health in rats during ages that are equivalent to humans in the neonatal and late infancy period. Specifically, we sought to develop and pilot a new biobehavioral assay for a rat model that mirrored the human infant General Movement Assessment.

## 2. Materials and Methods

### 2.1. Animals

All experiments were performed in strict accordance with protocols approved by the Institutional Animal Care and Use Committee (IACUC) at the Johns Hopkins University School of Medicine (RA21M362, PI: Jantzie, Lauren, Approval 19 July 2022). Protocols were developed and performed consistent with National Research Council and ARRIVE guidelines [17]. Sprague Dawley rat dams and litters purchased from Charles River Laboratories (Wilmington, MA, USA) were maintained in a temperature- and humidity-controlled facility with food and water available ad libitum. Animals were subject to 12 h (h) dark/light cycle, with lights on at 08:00 h.

### 2.2. In Utero Chorioamnionitis (CHORIO)

To induce a CP-like injury in rats, pregnant Sprague Dawley rats underwent abdominal laparotomy on embryonic day 18 (E18) to induce CHORIO as previously reported [14,15,18,19,20,21,22,23,24,25]. Pregnant rats were induced with 3.0% isoflurane. Via laparotomy, the uterus was externalized, and the uterine arteries were temporarily occluded with aneurism clips to induce transient placental insufficiency. After 60 min, clips were removed and intra-amniotic injection of lipopolysaccharide (LPS 0111: B4, 4 μg/sac; Sigma-Aldrich, St. Louis, MO, USA) was administered to each sac, and the laparotomy was closed. The rat pups were born at term on embryonic day 22 (E22). Sham animals had a laparotomy (no uterine artery occlusion nor LPS) with equivalent duration of anesthesia. For each experiment described, data represents true n (individual rats) from at least 3 different dams per condition. Male and female offspring were used in every outcome measure. Specifically, twenty-six rats, including 6 sham (4 females and 2 males) and 20 CHORIO/CP (11 females and 9 males) were included in this study. Previously, we have shown that this model yields cerebral palsy-like deficits in the mature CNS that mimic those of preterm survivors, with white matter injury, gliosis, spastic-like gait, poor social interaction, and cognitive impairment [14,15,18,19,20,21,22,23,24,25]. This model encompasses the fetal and systemic inflammatory response syndromes and placental pathology common in preterm infants with CHORIO.

### 2.3. General Movements Assessment in Rats (GMA)

**Figure 1:**
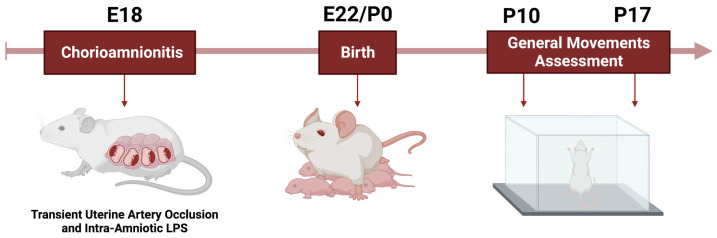
Experimental Design. CP-like injury was induced at embryonic day 18 (E18). Rat pups were born on embryonic day 22. Rats were placed in a cage on postnatal day 10 (P10) and postnatal day 17 (P17) and videoed. The videos were reviewed by a non-blinded evaluator for differences in sensory motor behaviors in the shams and rat pups with CP-like injury. A blinded evaluator then observed and coded for the specific behaviors.

In preparation for developing a behavioral coding scheme of rat motor behaviors, literature was reviewed about typical motor and sensorimotor development in rats [26]. On postnatal day 10 (P10, term equivalent age) and again on P17 (toddler equivalent age), 5 min videos were recorded using AnyMaze 6.3 software of each rat moving freely in a clean, contained, and well-lit (130 lm) cage. A neuropsychologist (GG), with training in coding of behaviors and who was certified in the human General Movements Assessment (GMA) that was not blind, reviewed all the animal videos at each age (shown in Figure 1). This is consistent with general practice for developing behavioral coding schemes in the practice of psychology [27]. Specifically, the behaviors, or general movements, were spontaneous movements that involved the rat pups’ whole body, neck, trunk and upper and lower extremities. Similar to what is observed in humans, these movements of the whole body that allowed sensory information to be obtained by the whiskers and movements that have been documented to be developmentally appropriate motor movements of the extremities [26] that include walking and rearing. Multidimensional nose sweeping was defined as movement of the head with sniffing in and outside of the body axis. Rearing was defined as time in seconds of alternating, independent movements to climb walls of the cage with forelimbs (paws wall with alternating limbs). Variability, jerking, circling, degree of exploration, and time immobile was also observed. The neuropsychologist/non-blinded reviewer identified two behaviors that differed between rat pups in each group, including multi-dimensional nose sweeping and sniffing at P10 and frequency of rearing at P17. Subsequently, each video was then scored for these criteria by an evaluator blinded to group status using AnyMaze 6.3 software in order to determine whether it discriminated between the two groups.

### 2.4. Statistical Analyses

Differences between 6 sham (4 females and 2 males) and 20 CHORIO/CP (11 females and 9 males) were analyzed using a Mann–Whitney U-test or Student’s *t*-test (*p* < 0.05 level of significance).

## 3. Results

### 3.1. Group Comparison at Postnatal Day 10

**Figure 2:**
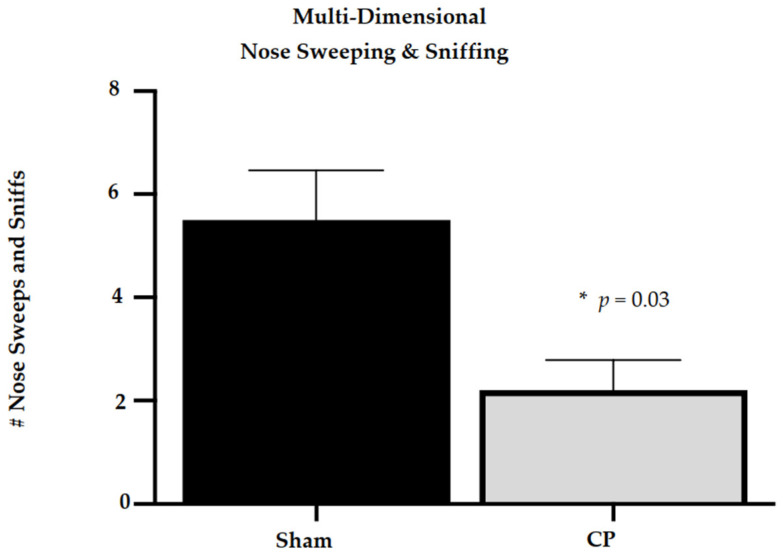
Multi-dimensional nose Sweeping and sniffing behaviors of neonatal rats (P10) with CP compared to sham controls. Neonatal rats with CP (N = 20) show less multi-dimensional nose sweeping and sniffing compared to sham control neonatal rats (N = 6). * *p* ≤ 0.05; # = number.

Neonatal rats at P10 in the CP group demonstrated decreased spatial explorations using multi-dimensional nose sweeping and sniffing compared to shams. There was a statistically significant difference in the number of episodes of nose sweeping in the CP group (2.2 ± 0.58 episodes) compared to the sham controls (5.5 ± 0.96 episodes), *U*(*N*_cp_ = 20, *N*_sham_ = 6) = 5.500, *p* = 0.03 (shown in Figure 2).

### 3.2. Group Comparison at Postnatal Day 17

**Figure 3:**
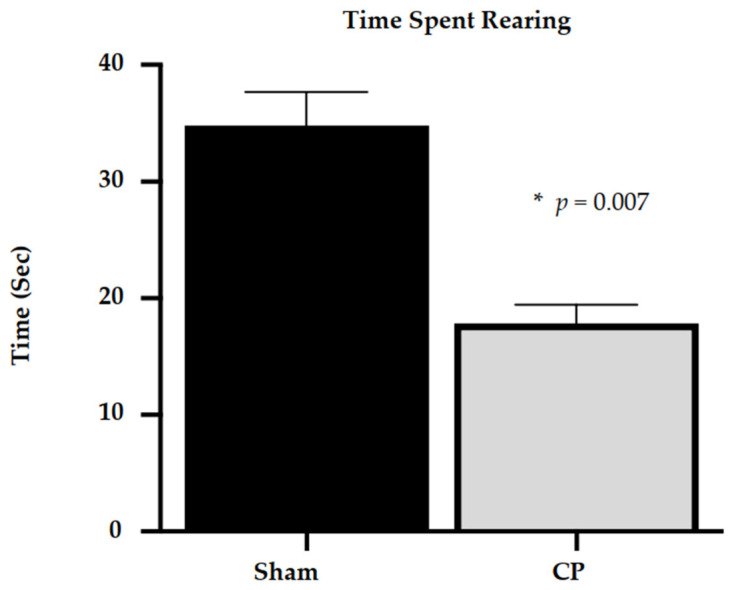
Rearing behaviors of rats in late infancy (P17) with CP compared to sham controls. Rats in late infancy with CP (N = 20) spend less time rearing compared to sham control neonatal rats (N = 6). * *p* ≤ 0.05.

At P17 (equivalent to late infancy in humans), rats with CP also exhibited sensorimotor impairment and decreased spatial exploration. Specifically, there was a statistically significant difference in the amount of time CP rats spent rearing (17.85 ± 1.60 s) compared to sham controls (34.8 ± 2.89 s), *t* (10) = 8.104, *p* = 0.007 (shown in Figure 3).

## 4. Discussion

Prechtl’s General Movement Assessment in human infants examines motor movements that are spontaneously generated motor movements involving the whole body that present in a variable sequence and evolve over the first 5 months of the human infant’s life [7]. These movements are theorized to occur because the sensorimotor system is recalibrating from the antigravity in utero environment to the extrauterine environment. The presence of the specific writhing and fidgety movements in human infants are therefore a foundation for later complex and precise volitional motor movement and without this foundation, motor impairments such as cerebral palsy (CP) are observed [3,6,7]. Research into the developmental milestones and behavior of rats has interesting parallels to humans, such that there is also an association between the presence of specific sensorimotor behaviors and subsequent typical or atypical sensori-motor development. More specifically, Smirov and Stinikova [28] demonstrated that trimming a rat’s whiskers in the neonatal period led to delays in all locomotor milestones. Therefore, like human infants engaging in specific patterns of non-volitional movement to learn about where their bodies are in space in the extrauterine environment, rats also engage in their own unique movements that allow for sensory input through their whiskers to create a foundation for later complex and precise motor movements. Furthermore, it is plausible that sensorimotor behaviors involving movements of rats’ snouts in the early postnatal period may be absent if there is brain injury acquired in the embryonic period, similar to the alterations or absence in spontaneously generated motor movements in human neonates who have perinatal brain injury following preterm birth, hypoxic-ischemic encephalopathy, or perinatal stroke.

The present pilot study provides some data that there are rat age specific movements of neurodevelopmental health (i.e., multi-dimensional sweeping of the nose and rearing) that correlate to equivalent human infant motor movements assessed on the General Movements Assessment over the first 5 months of life. Decreased frequency or absence of specific sensorimotor behaviors in rats appear to predict the risk of manifesting CP-like symptoms in rats similar to how specific sensorimotor behaviors in human infants predict the risk of CP. Specifically, the frequency of multi-dimensional nose sweeping and sniffing in neonatal rats are predictive of neurologic injury and CP-like symptoms by adulthood in rats, similar to the writhing movements observed in human neonates and characterized by the General Movement Assessment. Additionally, much like the absence of normal fidgety movements on General Movement Assessment are further predictive of CP risk in human infants within the first 3–5 months of life, reduced amounts of engagement in early rearing behaviors in rats at P17 is also predictive of CP-like symptoms.

### 4.1. Implications

The rat model of CP utilized in this study has previously provided a great deal of information about the mechanisms of injury underlying the behavioral manifestations of CP by adulthood, including motor and cognitive deficits [14,15,16,19]. This model captures both processes of hypoxic–ischemic and infectious inflammatory injury commonly observed among infants born preterm, and resulting in patterns of both acute and chronic white matter injury. The CP-like motor deficits commonly observed by adulthood in rats include an ataxic gait with specific deficits in stride, paw placement, gait consistency and coordination on Digigait assessment, which is consistent with motor deficits in older children and adult humans with CP [14,15].

Presently, clinical practice guidelines for diagnosis of CP have shifted, placing an important emphasis on the early detection of CP in humans (i.e., under the age 12 months in many cases; [1,2,4,5]). The General Movements Assessment is one specific tool that is important for early detection of CP in humans [6,7]. The development of an analogous tool in basic science is therefore critical in order for truly translational research in populations at high risk for diagnosis of CP. Ultimately, the development of analogous assessment tools is necessary to provide the most accurate and precise basic science approaches and the foundation for the development of treatments and interventions that can move more seamlessly from bench to bedside.

Beyond the development of translational motor assessments, the use of analogous measures of cognition is also critical, as similar cognitive-behavioral deficits have also been appreciated in humans, with brain injuries leading to CP in humans and CP-like motor impairments in rats. These cognitive-behavioral deficits include fewer social interactions compared to shams and deficits in visual discrimination and reversal learning on touchscreen operant chamber testing. Such cognitive-behavioral findings are in line with deficits in selective visual attention, and executive dysfunction, including problems with behavioral initiation and inhibition, as well as cognitive flexibility that are frequently observed among humans diagnosed with CP [16,19]. As such, increased harmonization of human and rat measurement tools to quantify these cognitive behavioral deficits will also be necessary, and the present study demonstrates the feasibility of this endeavor as a future direction for research.

### 4.2. Future Directions

In the future, additional examination of inter-rater reliability and external validity is warranted. It is critical that following training, there is a high level of consistency between evaluators for the coded behaviors. Further examination of these methods across multiple samples is also necessary to assure the results are generalizable across future studies and clinical trials.

Beyond the development of translational motor assessments, the use of analogous measures of cognition is also critical, as similar cognitive-behavioral deficits have also been appreciated in humans with brain injuries leading to CP in humans and CP-like motor impairments in rats. These cognitive-behavioral deficits include fewer social interactions compared to shams and deficits in visual discrimination and reversal learning on touchscreen operant chamber testing. Such cognitive-behavioral findings are in line with deficits in selective visual attention, and executive dysfunction, including problems with behavioral initiation and inhibition, as well as cognitive flexibility that are frequently observed among humans diagnosed with CP [16,19]. As such, increased harmonization of human and rat measurement tools to quantify these cognitive behavioral deficits will also be necessary, and the present study demonstrates the feasibility of this endeavor as a future direction for research.

## 5. Limitations

Limitations to the current study included the small sample size of our control group, and the reliability and validity of these methods were not fully explored. Concerning reliability and validity, the specific animal behaviors were only coded by one blinded evaluator, limiting the information regarding inter-rater reliability. Additionally, these methods were not examined across multiple samples, which currently limits the external validity.

## 6. Conclusions

Harmonization of clinical and preclinical methodologies is critical for facilitation of translational work, particularly in the area of perinatal brain injury as the field moves towards implementation of clinical trials. The gold standard measurement of CP-similar motor behaviors in adult rats has been successful using Digigait; however, with the push for early diagnosis of CP, diagnosis routinely occurs before 1 year of age and often as early as infancy. The lack of early diagnostic tools in rats that mirror the tools used in clinical practice and the incongruity in animal and human outcome models makes it much more challenging to develop translational studies and clinical trials that specifically target and evaluate therapeutic interventions within the neonatal period. In a search to identify early models for better translational work, we identified two behavioral biomarkers that differentiated brain injured rats from shams at early ages. Nose sweeping and sniffing at P10 (early infancy) and time spent rearing at P17 (late infancy) both identified rats who were at a high risk of CP in adulthood, similar to Prechtl’s General Movement\Assessment in terms of infants and at a 3-month corrected age. Our pilot findings of harmonized translational and precision biobehavioral assays provides an opportunity for increased expediency of clinical trials at the earliest stages of brain development, when there is an opportunity to mitigate injury that may result in a later diagnosis of CP.

## Data Availability

The dataset on which this paper is based is a very small pilot data sample and it is not retained or publicly archived with available resources.

## Abbreviations

ARRIVE	Animal Research: Reporting of In Vivo Experiments
CHORIO	Chorioamnionitis
CP	Cerebral palsy
CPARF	Cerebral Palsy Research Alliance Foundation
GMA	General Movements Assessment
Hr	hour
IACUC	Institutional Animal Care and Use Committee
LPS	lipopolysaccharide
NIH	National Institutes of Health
P	Postnatal day
